# Viruses of the Nahant Collection, characterization of 251 marine Vibrionaceae viruses

**DOI:** 10.1038/sdata.2018.114

**Published:** 2018-07-03

**Authors:** Kathryn M. Kauffman, Julia M. Brown, Radhey S. Sharma, David VanInsberghe, Joseph Elsherbini, Martin Polz, Libusha Kelly

**Affiliations:** 1 Department of Civil and Environmental Engineering, Massachusetts Institute of Technology, Cambridge, MA 02141 USA; 2 Department of Systems and Computational Biology, Albert Einstein College of Medicine, Bronx, NY 10461 USA

**Keywords:** Mobile elements, Marine biology, Virology, DNA sequencing

## Abstract

Viruses are highly discriminating in their interactions with host cells and are thought to play a major role in maintaining diversity of environmental microbes. However, large-scale ecological and genomic studies of co-occurring virus-host pairs, required to characterize the mechanistic and genomic foundations of virus-host interactions, are lacking. Here, we present the largest dataset of cultivated and sequenced co-occurring virus-host pairs that captures ecologically representative fine-scale diversity. Using the ubiquitous and ecologically diverse marine Vibrionaceae as a host platform, we isolate and sequence 251 dsDNA viruses and their hosts from three time points within a 93-day time-series study. The virus collection includes representatives of the three *Caudovirales* tailed virus morphotypes, a novel family of nontailed viruses, and the smallest (10,046 bp) and largest (348,911 bp) *Vibrio* virus genomes described. We provide general characterization and annotation of the viruses and describe read-mapping protocols to standardize genome presentation. The rich ecological and genomic contextualization of hosts and viruses make the Nahant Collection a unique platform for high-resolution studies of environmental virus-host infection networks.

## Background & Summary

Viruses influence the structure, function, ecology, and evolution of microbial communities. They represent the richest reservoir of nucleic acid diversity^1^, and the great abundance of viral particles in the environment^1^ reflects the expression of these sequences in host cells^2^. However, understanding the structure of virus-host interaction networks in the wild still poses a major challenge as pair-wise interactions between specific viruses and their hosts cannot be predicted without isolate based studies. Thus, though viruses have been predicted to play a major role in maintaining the extensive fine-scale genomic diversity of environmental microbes, it has not been possible to systematically evaluate the mechanistic or genomic foundations of these interactions, nor their ecological and evolutionary consequences.

Here, we present the annotated viral genomes of the Nahant Collection, a large-scale virus-host model system of cultivated and genome-sequenced bacterial and viral isolates, built on the extensively characterized environmental marine Vibrionaceae model system. By capturing large numbers of closely related host and virus strains, the Nahant Collection allows evaluation of the impact of ecologically relevant fine scale diversity on the interactions between bacteria and lytic viruses. This collection of 251 virus genomes and their associated pairs is a resource for interrogating the determinants of host range and the molecular bases of specific virus-host interactions within one of the most richly contextualized environmental microbial model systems^3–7^ and time series studies^8^ available.

Viruses and hosts were isolated from samples collected at three time points within a 93-consecutive-day study of littoral marine microbial communities, the Nahant Time Series^8^. All viruses were isolated on hosts collected on the same day, and hosts were nearly exclusively *Vibrio*. The *Vibrio* are a well-suited host group for the evaluation of the role of ecology and evolution in structuring virus-host interactions – they are ubiquitous in marine systems, ecologically diverse, and are among the most thoroughly characterized model systems for the study of bacterial populations in the wild^3–6,9,10^. The viruses were recovered using approaches designed to yield representation of diverse viruses, including: isolation from concentrates by plating in agar overlays to allow for more representative recovery of both fast- and slow-growing viruses; use of 2-week incubation times to allow for appearance of plaques by slow plaque-formers; and inclusion of additives to media to improve plaque visualization (glycerol^11^) and mimic environmental substrates (chitin) that might be necessary to induce expression of host receptors.

To standardize assemblies of purified viral isolate genomes we used an approach informed by predicted differences in packaging strategies among viruses, described in greater detail in the methods. This approach suggests that viruses of the Nahant Collection include members with diverse packaged genome types, including cohesive end overhangs, inverted terminal repeats, headful-packaging type terminal redundancy, and Mu-like host ends. To evaluate whether any of the viruses were prophages derived from the host of isolation, rather than environmentally-derived isolates, sequence-based searches between virus and isolation host genomes were performed; only one case of prophage purification was identified among the virus strains with sequenced host genomes (Supplementary Table 1).

**Figure 1:**
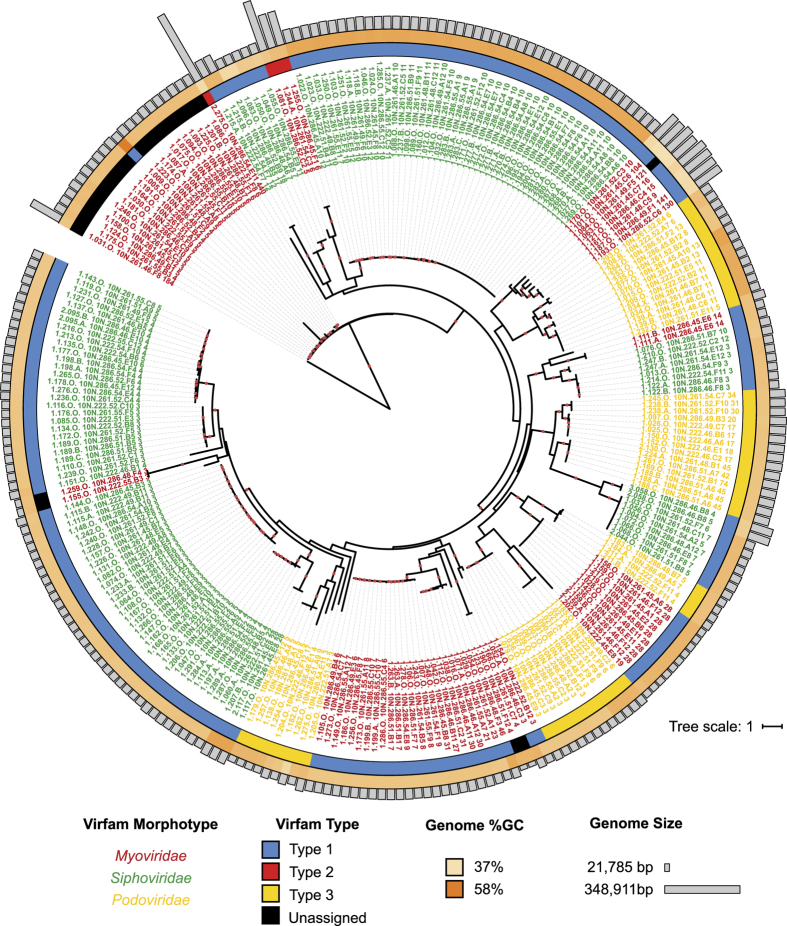
Overview of the diversity of Nahant Collection tailed viruses, organized by portal protein phylogeny. Virfam^12^ classifier annotation of the Nahant Collection *Caudovirales* viruses reveals a diverse collection of myo-, sipho-, and podoviruses (indicated by color of leaf lable) representing all Types and Clusters (indicated in first attribute ring, and see Discussion for cluster identifiers) previously known to infect Proteobacteria, as well as many genomes unassignable to previously described groups. All 262 *Caudovirales* genome sequences are presented, including 28 replicate sub-lineage genomes. Genome %GC is provided in the second attribute ring and genome size is indicated by bars. The portal protein tree is unrooted and based on trimmed alignments; red circles indicate aLRT-supports ≥0.9. Associated data provided in Table 1, portal protein sequences in provided in Supplementary Table 3, interactive tree available at http://itol.embl.de/tree/181897146181191519509155#.

The collection includes highly diverse dsDNA tailed and non-tailed viruses, including the smallest (10,046 bp) and largest (348,911 bp) described *Vibrio* virus genomes (median 45,072 bp). Using the Virfam^12^ *Caudovirales* classifier, we find that the tailed viruses include representatives of all Virfam Types and Clusters previously identified as associated with Proteobacteria, including: Type 1 Clusters 3, 5, 6, 7, 8, 9 (*Siphoviridae* and *Myoviridae*); Type 2 (*Myoviridae*); and Type 3 (*Podoviridae*), as well as 26 viruses not associated to any previously identified Virfam Types or Clusters. Analyzing portal protein phylogeny revealed groups of closely related viruses as well as extensive collection-wide portal protein diversity (Fig. 1). The non-tailed viruses discovered in the collection are a proposed novel family, the *Autolykviridae*, and are discussed in greater detail elsewhere^13^. The overall collection ranges from 37% to 58% GC content, with a median of 43%. Viral genomes in the collection are also notable for their carriage of tRNAs, present in 53 viruses, and the presence of putative CRISPR features, present in 32 viruses (Table 1 (available online only)).

The viruses and hosts of the Nahant Collection, the largest available dataset of sequenced co-occurring cultivated virus-host pairs, are embedded within the rich contextualization of the 93-consecutive-day Nahant Time Series study^8^. The integration of ecological context, sequence-information, and cultivation-based study available for this model system make the Nahant Collection a unique and robust foundation for the study of the role of viruses in the ecology and evolution of their bacterial hosts.

## Methods

### Environmental sampling

All viruses and their hosts were isolated from water samples collected at three time points within a larger 3-month study^8^ of coastal marine microbial communities at Canoe Cove, Nahant, MA, USA in 2010 (42° 25’ 10.6”N, 70° 54’ 24.2”W): August 10 (ordinal day 222, water temperature 13.8 °C), September 18 (261, 16.3 °C), and October 13 (286, 14.2 °C). Bacteria were collected using a size-fractionation approach^3,4^ designed to partition co-occurring strains on the basis of differential associations in the water column. Here, as in previous studies of the *Vibrio*^3,4^, bacteria were isolated by dilution series plating of material resuspended from 63 μm, 5 μm, 1 μm, and 0.2 μm size-fractions onto vibrio-selective media (MTCBS) for colony growth and serial purification. We purified 3,456 bacterial isolates comprised of 1,152 strains from each of 3 days, evenly distributed over the size-fractions. Samples collected for isolation of viruses were 0.2 μm-filtered to remove bacteria, flocculated by addition of iron chloride, and the flocs collected on 0.2 μm filters and re-dissolved in oxalate for storage at 4˚C (ref. 14). Using this approach, viable viruses in 1000x-fold concentrated seawater could be preserved for later isolation on bacterial isolates derived from the same time and place.

### Agar overlay direct plating of concentrates for isolation of viruses

To isolate viruses on co-occurring hosts we used a quantitative agar-overlay approach that allowed for equal representation of both slow- and fast-growing viruses as follows. Viral concentrates equivalent to 15 ml of seawater (15 μl iron-oxalate concentrate) were mixed with host cultures to form agar-overlays within which discrete plaques could form and from which viruses could be isolated^15^. In total 1,334 purified bacterial strains were exposed, comprising >400 strains per isolation day and representing all isolation size-fractions; of these, 295 showed plaques. Agar overlays were performed using 150 μl of host overnight culture, 2 ml of molten top agar (52 °C, 0.4% agar, 5% glycerol, in 2216 marine broth [MB]), and bottom agar containing glycerol and chitin (1% agar, 5% glycerol, 125 ml L^−1^ of chitin supplement [40 g L^−1^ coarsely ground chitin, autoclaved, 0.2 um filtered] in 2216 MB). Glycerol was added to increase the visibility of plaques^11^, chitin was added to increase the probability of recovery of viruses dependent on chitin-induced receptors, and low density top agar was used to increase the probability of plaque formation by larger viruses^16^. Agar overlays were wrapped with plastic to reduce desiccation and held at room temperature for 14-16 days. Virus plaques were harvested at the end of the incubation period and archived by filtration of plaque eluates, as described in (ref. 15). Half of each eluate was stored at 4˚C, and half was preserved with glycerol (to a concentration of 25% glycerol) for storage at −20˚C.

### Purification of viral strains

To build a diverse and representative collection of virus-host pairs, at least one randomly selected virus was purified from each bacterial strain for which plaques appeared in the agar overlay plating of environmental concentrate. To achieve this, we serially purified viruses recovered from archived material, prepared small-scale lysates to boost viral titer, and then generated high titer stocks by confluent lysis in agar overlays. Purification resulted in genome sequencing of 283 viral strains (from 251 independent plaques) from 246 hosts, described below. Viral and host strain naming conventions are described in Table 2, using examples of virus 1.008.O_10 N.286.54.E5 and host 10N.286.54.E5.

### Genome sequencing

Viral genomes were prepared from lysates of the host of isolation, as follows. Lysates were concentrated on centrifugal filtration devices (Ultracel 30 K, Amicon Ultra, Millipore, UFC903024), washed with 1:100 2216MB, and concentrates treated with nucleases to digest unencapsidated nucleic acids (18 ml sample brought to 500 μl and amended with DNase I, RNase A, heated for 65 min at 37˚C). Nuclease-treated samples were extracted by addition of 0.1 final volume of SDS mix (0.25 M EDTA; 0.5 M Tris-HCl, pH 9.0; 2.5% sodium dodecyl sulfate), 30 min incubation at 65˚C, addition of 0.125 volumes 8 M potassium acetate, 60 min incubation on ice, addition of 0.5 volumes of phenol-chloroform, and recovery of nucleic acids from aqueous phase by isopropanol and ethanol precipitation. Illumina sequencing libraries of each extract were prepared as follows. Sample DNA (5 μg in 100 μl) was sheared by sonication (6 cycles of 5 min each at an interval of 30 sec on/off on the ‘Low Intensity’ setting of the Biogenode Bioruptor) to enrich for fragment sizes of ~300 bp. Sequencing constructs were prepared by end repair of sheared DNA, 0.72x/0.21x dSPRI size selection to enrich for ~300 bp sized fragments, ligation of Illumina adapters and unique pairs of forward and reverse barcodes for each sample, SPRI bead clean-up, nick translation, and final SPRI bead clean-up^17^. Constructs were enriched by PCR using paired-end (PE) primers following qPCR-based normalization of template concentrations. Enrichment PCRs were prepared in eight replicate 25 μl volumes, with the recipe: 1 μl Illumina construct template, 5 μl 5x Phusion polymerase buffer, 0.5 μl 10 mM dNTPs, 0.25 μl 40 μm IGA-PCR-PE-F primer, 0.25 μl 40 μm IGA-PCR-PE-R primer, 0.25 μl Phusion polymerase, 17.75 μl PCR-grade water. PCR thermocycling conditions were as follows: initial denaturation at 98 °C for 20 sec; batch dependent number of cycles of 98 °C for 15 sec, 60 °C for 20 sec, 72 °C for 20 sec; final annealing at 72 °C for 5 min; hold at 10 °C. For each sample 8 replicate enrichment PCR reactions were pooled and purified by 0.8x SPRI bead clean-up. Each sample was then checked by Bioanalyzer (2100 expert High Sensitivity DNA Assay) to confirm the presence of a unimodal distribution of fragments with a peak between 350-500 bp. Sequencing of viral genomes was distributed over 4 paired-end sequencing runs as follows: 1 lane on the Illumina HiSeq2000 (18 viral genomes; 100+100 nt paired-end reads; average of 5.1 million reads per genome), 3 lanes on the Illumina MiSeq (92-96 genomes per lane; 150+150 nt paired-end reads; average of 54 K, 208 K, 210 K reads per genome for each lane). Raw paired-end Illumina reads were imported and demultiplexed using CLC Genomics Workbench v.6.5.1 (https://www.qiagenbioinformatics.com/). Sequencing and assembly of genomes of bacterial hosts is described elsewhere^13^.

### Genome assembly and curation

Differences in packaging strategies among viruses yield distinctive and characteristic distributions of packaged physical genomes in progeny virions^18^. Common examples of such strategies include production of virions with genomes that have: variable termini comprised of host DNA (Mu-like viruses); 5′ or 3′ single strand terminal overhangs (cos-viruses); or different start sequences and terminal redundancies ranging from 10 s to 10,000 s of bases (pac-viruses). To inform final curation of genome sequences, we first performed initial assemblies to group similar genomes and allow identification of the packaging-associated large subunit terminase gene (TerL) where possible. We then evaluated read mapping profiles within groups, considering terminase-predicted packaging strategy, to define final genome start sites. We next used an iterative approach, as described below, to standardize genome assemblies with conserved gene orders and genomic start positions for related viruses, and to place genomic termini at the contig ends.

### Initial assembly and viral genome clustering

Initial assembly and clustering of viral genomes identified groups of related viruses (Supplementary Table 2), but also highlighted the need for systematic measures to standardize genome curation. Initial assembly and clustering were performed as follows: viral genomes were assembled using the *de novo* assembly tool in CLC Genomics WorkBench v.6.5.1 with default parameters following trimming of reads (default parameters except: quality score=0.01, ambiguous nucleotides=0). Open reading frames (ORFs) were identified using Prodigal^19^ with default parameters, and reciprocal best BLAST hits with ≥75% coverage of the longer sequence and e-value of ≤10^−5^ were clustered using OrthoMCL^20^. Viral genomes were clustered into genome groups on the basis of shared protein clusters using the FT algorithm of the ClustnSee^21^ plug-in in Cytoscape^22^. Preliminary curation of individual groups to assess synteny between closely related and replicate viruses (see Technical Validation) using LAST^23^ indicated that assemblies began at different locations, suggesting that virus genome characteristics were confounding consistency in contig start and end sites.

### Final assembly and curation

To systematically address the inconsistency in contigs produced by assemblies of closely related viruses, assemblies were repeated and curated based on read mapping patterns and terminase similarities, as described below.

Viral genomes were re-assembled using the command clc_assembler from CLC Assembly Cell (version 4.4.2, https://www.qiagenbioinformatics.com/), using default assembly parameters and an insert size setting of 100 to 300 bp; 154 out of 285 assemblies resulted in one contiguous sequence (contig). For virus assemblies producing more than one contig, the highest coverage contig was extracted and considered the target viral genome contig; lower coverage contigs were considered contamination from host genome or prophages.

**Figure 2:**
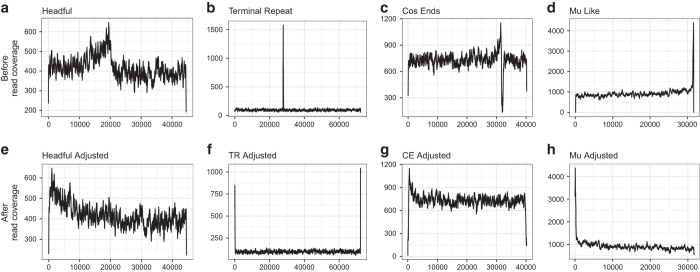
Examples of read recruitment by contig assemblies before and after adjustment for virus genomes with differing read mapping patterns. Coverage mapping onto contigs of viruses with: Headful-like read mapping before (**a**) and after contig adjustment (**e**); Terminal repeat-like read mapping before (**b**) and after (**f**) contig adjustment; Single-stranded cos-end-like read mapping before (**c**) and after (**g**) contig adjustment; Mu-like read mapping before (**d**) and after (**h**) contig adjustment. Note that, as indicated in the methods, though read mapping patterns were evaluated for each virus, final adjustment strategy for each virus (Supplementary Table 2) was not determined solely based on read mapping pattern and the majority of virus contigs were defined as starting one open reading frame upstream of the large subunit of the terminase (TerL) regardless of the read mapping pattern.

Viral genome open reading frames (ORFs) were identified using Prodigal version 2.6.1 with the -p meta flag to identify small ORFs^24^, and virus terminase protein sequences were identified by UBLAST^25^ search with a cutoff evalue<0.001 against a database of terminases from public viral genomes with previously described or predicted physical genomic termini^18,25^. Terminase identity was initially assessed via UBLAST as described above, and then verified via OrthoMCL clustering of terminase ORFs with the same dataset to gauge the fidelity of the BLAST results. To evaluate read coverage patterns in relation to terminases, original reads were mapped back to the contigs using the clc_mapper command with default parameters and per-base coverage was determined using SAMtools^26^ and BEDtools^27^. Consistent with previous findings that different terminases are associated with distinct genome packaging strategies^18^, and thus genome termini, exploratory evaluation of read coverage patterns showed that viruses with close identity to different known phage terminases also generally showed different read coverage patterns (Fig. 2a–d).

To standardize final gene order presentations, start and stop positions for each genome were defined manually, considering three criteria: 1) terminase identity, as identified by UBLAST and OrthoMCL clustering; 2) read coverage; and 3) comparison of contigs between viruses within the genome groups identified in the initial assembly. All members of each group were assigned to a common inferred genome packaging strategy category (see Supplementary Table 2 for details and exceptions) on the basis of overall patterns within the group, and rearranged using the approaches described below. Coverage patterns were determined by visual inspection and by a series of custom R scripts. Where possible, finalized virus genomes were quality checked by comparing the synteny of related phage genomes using command line LAST. A total of 283 virus genomes were assembled, including 251 unique viruses, 31 sub-lineages purified in parallel to the primary unique isolate, and 1 technical replicate (Table 1 (available online only)). We note that though we were guided by group-level coverage patterns, our primary aim was standardization rather than inference of true genome topology, which must be defined by individual genome read coverage patterns and complemented by laboratory studies.

#### Re-arrangement based on specific ORF

The majority of viral genomes (168/283) in the collection were standardized by circularizing the *de novo* assembled contigs and re-linearizing them at the start of the ORF upstream of the terminase. As a whole, these viruses showed coverage patterns consistent with a headful, or ‘pac’ site, based genome packaging strategy, wherein up to 110% genome-length monomers are sequentially cleaved from a multigenome-length concatemer, beginning from a conserved ‘pac’ site. Terminase best BLAST matches were dominated by similarity to viruses with headful-like strategies (Sf6, 97 best hits; 933W, 12; and T4, 5), though best hits to short direct terminal repeat (T7, 8) and 5′-cohesive ends (P2, 26) viruses were also identified, along with cases of no similarity to reference virus terminases (20). Read coverage patterns among these viruses were dominated by either a pattern of gradual decreases/shifts (112) consistent with a ‘headful’ or packaging site (‘pac’) – based genome packaging strategy, or even coverage (46); though other patterns of coverage including short peaks (cos pattern, 1; short internal peak, 7) and multiple coverage peaks (2) were also observed. Examination of read coverage following TerL-based rearrangement of contigs (Fig. 2e) often showed coverage maxima localized near the start, consistent with previous observations that headful-packaging viruses commonly have a pac site in or near the small subunit of the terminase gene^18^, which generally lies upstream of the TerL. Viruses curated using this approach included all the viruses from 7 of the preliminary groups (1, 4, 6, 9, 10, 13, 16), the majority of viruses from group 3, and a single virus from group 7.

#### Re-arrangement based on peaks or valleys in coverage

The second most commonly applied strategy for standardization (66/283) was circularization of contigs followed by re-linearization by cutting in the middle of a short region of either aberrantly low (36), or high (30), coverage (Fig. 2f,g). As a whole, these viruses showed patterns consistent with the presence of either direct terminal repeats (DTRs) or single-stranded cohesive (‘cos’) ends associated with their genome termini. Genomes with DTRs may yield sharply defined regions of elevated coverage. ‘cos’ genomes may yield regions of either high or low coverage, depending on whether they are 3′ or 5′ overhangs, due to low frequency ligation of ends during library preparation, as well as T4 DNA polymerase 3′ to 5′ exonuclease activity (degradation of 3′ overhangs) and 5′ to 3′ polymerase activity (endfill of 5′ overhangs) of unligated ends. Terminase best BLAST matches were dominated by similarity to viruses with cohesive ends (lambda ‘cos’, 22; HK97 ‘cos’-3′, 7; P2 ‘cos’-5′, 3) and DTRs (N4, 8; T7, 13), though best hits to headful viruses (933W, 4; P22, 1), were also identified, along with cases of no similarity to reference virus terminases (8). Read coverage patterns among these viruses were dominated by either distinctive ‘cos’ (32) or short internal peak (22) patterns, though other patterns of coverage including shifts in coverage (8), multiple coverage peaks (2), even coverage (1), or no pattern (1) were also observed. This approach included all virus genomes in preliminary groups 8, 14, and 18; the majority of viruses in groups 5, 7, 11, and 12; and a minority of viruses in groups 3 and 17.

#### Scaffolded assembly against reference

If viruses did not follow the patterns described above, but closely related members of the same group (identified as sharing 100% of translated proteins identified via reciprocal UBLAST) did follow a particular pattern, viruses were assembled using closely related strains as a scaffold; this approach was used for genomes in group 5 (3).

#### Maintenance of original de novo assembly

Singleton viruses with no similar members within the dataset were treated based on closest terminase identity and read coverage pattern, but if no distinct pattern was observed the original assemblies were maintained. This approach was used for 16/283 viruses, including viruses in groups 2 (8), 3 (1), 7 (5), 11 (1), and 12 (1).

#### Removal of terminal unconserved sequences

A subset of viruses (9/283) were found by BLAST comparison to possess Mu-like terminases, suggesting that they also used a Mu-like replicative transposition headful mechanism that incorporates host DNA upstream and downstream of the site of insertion. Read coverage patterns of initial assemblies of Mu-like viruses exhibited sharp drops in coverage at the termini followed by regions of low coverage (Fig. 2d), these regions of low coverage, representing small pieces of the host genome, were removed in the adjusted assemblies (Fig. 2h). However, closer evaluation of these assemblies revealed several cases of truncated sequences and final Mu-like virus assemblies were performed in CLC Genomics Workbench 8.5.1 as follows. Sequences were trimmed using the NGS Core Tools Trim Sequences tool with trims based on quality scores (limit 0.0001), number of allowable ambiguous nucleotides (max 0), and discard of reads <50 bases. All remaining read pairs and orphans were assembled using the De Novo Assembly tool with a word size of 64 and otherwise default options. The largest contig was extracted from the assembly for each virus and all genomes were aligned using the Geneious 6.0.6 Map to Reference tool to standardize orientation. Genome termini were defined based on the beginning and end of conserved regions at the left and right genome ends, respectively. This yielded 9 independently isolated genomes that were all 100% nucleotide identical and with a length of 31,617 bases, with the exception of virus 1.159.O, which contained a single SNP that was present in both the new and previous assembly versions. Open reading frames for these genomes were called with Prodigal 2.6.3 using the -p meta flag and otherwise default parameters.

#### Iterative assembly

A subset of the viruses (21/283), described elsewhere as a new family^13^, had distinctively short (~10 kb) genomes and did not contain predicted terminases. BLAST comparison of ORFs from these genomes showed similarity to the protein-primed DNA polymerase of viruses of the *Tectiviridae*, which have linear genomes and inverted terminal repeats (ITR), and thus these viruses were also evaluated for ITRs. Final assemblies for this group were performed iteratively, as follows. Following initial assembly, second and third assembly iterations were performed using an increased word size of 64, and the largest contig from the previous assembly was included as one of the “reads” for the successive round of assembly. The longest contigs from each of the three assemblies were then compared and the longest contig that also exhibited ITRs was used, when none of the contigs contained ITRs the longest assembled contig was determined to be the final assembly.

### Annotation

Viral genomes were annotated using multiple approaches and tools, as described below. Genomes are available through Genbank (Data Citation 1).

#### Virfam classification of viral Types and Clusters and morphotypes

Viral proteins were annotated using the Virfam^12^ classifier, which identifies multiple genes of the head-neck-tail modules of viral genomes and assigns viral genomes to morphotypes within Types and Clusters on the basis of previous characterization of diverse tailed viruses. Annotation was performed individually per genome by submission to the Virfam webserver (http://biodev.cea.fr/virfam/). Output reports for all Virfam annotation runs are available through figshare (Data Citation 2).

#### Genome content annotation

Phage proteomes were compared to KEGG^28^, COG^29^, eggnog^30^, Pfam^31^, ACLAME^32^, CAMERA Viral Proteins (CVP)^33^ and the OM-RGC collection of sequences^34^ via UBLAST^24^. Annotations were determined as the best hit (maximum bit score) to a non-hypothetical protein from EggNOG, KEGG, COG, ACLAME or Pfam (minimum alignment of 75%, minimum percent identity of 35%). Best hits to remaining databases as well as CVP and OM-RGC are reported as notes within the final annotations. Annotations were combined with annotations identified using InterProScan version 5.17-56.0 using the iprlookup, goterms, and pathways options. InterProScan is a program from EMBL-EBI that uses the InterPro database for annotations. The InterPro database contains by default 13 databases, which are listed here: https://github.com/ebi-pf-team/interproscan/wiki/HowToRun#included-analyses. For these annotations, two optional databases were included: TMHMM for predicted transmembrane proteins and SignalP for predicted signal peptide cleavage sites. tRNA sequences were identified using tRNAscan-SE version 1.23 (ref. 35) using the general tRNA model (-G). CRISPR-like elements were identified using CRT^36^.

### Portal protein phylogeny

Portal proteins were identified directly using the Virfam classifier as described above, which provides a portal prediction, as well as by using HMM- and blastp-based searches of all Nahant Collection virus proteins, as follows. The portal protein for the representative virus of each Virfam cluster was downloaded through the Virfam page (http://biodev.cea.fr/virfam/), and an HMM generated by performing 3 iterations of Jackhmmer^37^ https://www.ebi.ac.uk/Tools/hmmer/search/jackhmmer. Searches of the Nahant Collection virus proteins with this collection of HMMs using the hmmsearch^38^ tool (hmmer version 3.1b2) identified putative portal proteins in 241 genomes, these 241 together with 6 portal proteins identified directly through the Virfam web page, were used to search the Nahant Collection with blastp^39^, identifying putative portal proteins in 262 of the 263 *Caudovirales* (e value <0.0001). In the 2 cases where the predicted portal proteins differed across the two methods (Supplementary Table 3), HHpred as implemented in the MPI bioinformatics Toolkit^40^ (https://toolkit.tuebingen.mpg.de/#/tools/hhpred) was used to evaluate both predictions and the protein with the longer sequence similarity to a portal protein was selected. The portal protein in virus 1.031.O could only be predicted using the Virfam approach and this protein was included, though HHpred and Phyre2 (ref. 41) based structural similarity based searches did not indicate similarity to known portal proteins. Sequence alignment, trimming, and tree-building were performed using the eggnog41 workflow in the ETE3 (ref. 42) version 3.0.0b36 tree building tool, the tree was visualized using iTOL^43^, and the figure prepared using Adobe Illustrator.

## Data Records

All virus and host-associated sequences and annotations associated with this work have been deposited to the Nahant Collection NCBI BioProject (Data Citation 1), specific accession numbers for each strain are provided in Supplementary Table 1. Viral genome annotation reports generated by the Virfam tool have been deposited with figshare (Data Citation 2).

## Technical Validation

Given the known abundance of prophages in bacterial genomes we evaluated whether any viruses in the collection represented induced prophages from the host of isolation. Using megaBLAST in Geneious 6.1.8 we searched all virus genomes against all sequenced hosts, we identified only a single case of a high query cover and high identity match. The virus 1.202.O (32,014 bp) shared a 30,051 bp 100% identity match with its host 10N.222.45.E8; this match region occurred within a larger host contig of 120,557 bp, suggesting that the failure to achieve a full match with the remaining 1,963 bp region of the virus genome contig was not due to incomplete assembly of the associated host region. Full genomes for the host of isolation were not available for 29 viruses and thus this prophage derivation could not be assessed for these strains, information on host sequence availability is provided in Supplementary Table 1.

This dataset contained sets of virus pairs and triplets that served as biological replicates for assembly optimization. Such sets derive from instances of independent purification of viral sub-lineages from a parent plaque due to the occurrence of variable plaque morphology. Though they exhibited sites of polymorphism at the nucleotide level, ranging from 0 to 4 SNPs, and indels of up to 201 bp (Table 3), members of these sets consistently showed identical gene content and are expected to have the same genomic structure and gene order. Methods for rearrangement to maintain synteny were developed around such sets and were verified via alignment of similar/duplicate genomes before and after rearrangement (Supplementary Figures 1–4).

## Additional information

**How to cite this article**: Kauffman, K. M. *et al*. Viruses of the Nahant Collection, characterization of 251 marine Vibrionaceae viruses. *Sci. Data* 5:180114 doi: 10.1038/sdata.2018.114 (2018).

**Publisher’s note**: Springer Nature remains neutral with regard to jurisdictional claims in published maps and institutional affiliations.

## Supplementary Material



Supplementary Information

Supplementary Table 1

Supplementary Table 2

Supplementary Table 3

## Figures and Tables

**Table 1:** Characteristics of Nahant Collection virus genomes

**Virus Name**	**Isolation host population**	**Coverage**	**Genome Length**	**GC%**	**Coding Sequences**	**tRNA**	**Putative CRISPR features**	**Virfam Morphotype**	**Virfam Type & Cluster**	**Is sub-lineage or replicate?**
Vibrio phage 1.003.O._10N.286.48.A2	*Vibrio lentus*	953	41,891	42.8421	72	0	0	Podo	Type-3	no
Vibrio phage 1.004.O._10N.261.54.A2	*Vibrio lentus*	25957	42,511	44.9837	50	0	0	Sipho	Type-1_Cluster-6	no
Vibrio phage 1.005.O._10N.286.48.F2	*Vibrio splendidus*	14049	50,301	43.9614	69	0	0	Myo	Unassigned	no
Vibrio phage 1.007.O._10N.261.55.F9	*Vibrio cyclitrophicus*	13070	49,244	42.8458	80	4	0	Myo	Type-1_Cluster-7	no
Vibrio phage 1.008.O._10N.286.54.E5	*Vibrio cyclitrophicus*	963	10,579	43.3311	21	0	1	.	.	no
Vibrio phage 1.009.O._10N.261.51.C9	*Vibrio lentus*	9690	44,443	57.6919	62	0	1	Myo	Type-1_Cluster-6	no
Vibrio phage 1.011.O._10N.286.49.B11	*Vibrio cyclitrophicus*	1461	10,579	43.3595	20	0	1	.	.	no
Vibrio phage 1.012.O._10N.261.48.C12	*Vibrio lentus*	8179	59,979	48.6070	98	1	0	Sipho	Type-1_Cluster-5	no
Vibrio phage 1.013.O._10N.286.54.F9	*Vibrio cyclitrophicus*	9139	44,457	43.8626	67	2	0	Sipho	Type-1	no
Vibrio phage 1.015.O._10N.222.51.E5	*Vibrio breoganii*	880	42,586	47.6119	66	0	0	Podo	Type-3	no
Vibrio phage 1.016.O._10N.286.46.A11	*Vibrio lentus*	7686	48,078	43.2880	89	0	0	Myo	Type-1_Cluster-7	no
Vibrio phage 1.017.O._10N.286.55.C11	*Vibrio sp. F12*	3333	51,290	44.2874	73	0	0	Myo	Unassigned	no
Vibrio phage 1.020.O._10N.222.48.A2	*Vibrio tasmaniensis*	405	10,636	43.6630	21	0	0	.	.	no
Vibrio phage 1.021.A._10N.222.51.F9	*Vibrio splendidus*	617	43,743	46.5172	50	0	0	Podo	Type-3	no
Vibrio phage 1.021.B._10N.222.51.F9	*Vibrio splendidus*	557	43,743	46.5172	50	0	0	Podo	Type-3	yes
Vibrio phage 1.021.C._10N.222.51.F9	*Vibrio splendidus*	625	43,743	46.5149	50	0	0	Podo	Type-3	yes
Vibrio phage 1.022.O._10N.286.45.A10	*Vibrio splendidus*	326	62,440	48.5554	97	1	0	Sipho	Type-1	no
Vibrio phage 1.023.O._10N.222.51.B4	*Vibrio splendidus*	170	59,872	48.6254	91	2	0	Sipho	Type-1	no
Vibrio phage 1.024.O._10N.261.45.F8	*Vibrio lentus*	5579	58,892	49.0610	83	1	1	Sipho	Type-1	no
Vibrio phage 1.025.O._10N.222.46.B6	*Vibrio splendidus*	637	75,797	43.1046	108	1	0	Podo	Type-3	no
Vibrio phage 1.026.O._10N.222.49.C7	*Vibrio splendidus*	200	75,880	43.1023	107	1	0	Podo	Type-3	no
Vibrio phage 1.027.O._10N.286.54.B8	*Vibrio lentus*	447	59,297	48.5927	85	0	0	Sipho	Type-1	no
Vibrio phage 1.028.O._10N.286.45.B6	*Vibrio sp.*	661	31,617	45.9436	50	0	0	Myo	Type-1_Cluster-8	no
Vibrio phage 1.029.O._10N.261.55.A7	*Vibrio sp.*	6525	46,513	42.5537	83	0	0	Myo	Type-1_Cluster-7	no
Vibrio phage 1.030.O._10N.222.55.F9	*Vibrio sp. F13*	776	45,827	43.8933	66	0	0	Myo	Unassigned	no
Vibrio phage 1.031.O._10N.261.46.F8	*Vibrio lentus*	247	152,942	43.1588	198	2	0	Myo	Unassigned	no
Vibrio phage 1.032.O._10N.261.54.F5	*Vibrio splendidus*	8522	60,399	48.4445	94	1	0	Sipho	Type-1	no
Vibrio phage 1.033.O._10N.222.49.B8	*Vibrio lentus*	7852	61,206	48.3531	89	1	0	Sipho	Type-1	no
Vibrio phage 1.034.O._10N.261.46.B7	*Vibrio breoganii*	795	44,398	48.0224	67	0	1	Podo	Type-3	no
Vibrio phage 1.034.X._10N.261.46.B7	*Vibrio breoganii*	821	44,383	48.0206	68	0	1	Podo	Type-3	yes, technical replicate
Vibrio phage 1.036.O._10N.286.45.C3	*Vibrio lentus*	17026	40,172	42.7985	62	0	0	Podo	Type-3	no
Vibrio phage 1.037.O._10N.261.52.F7	*Vibrio lentus*	563	43,111	44.9676	50	0	0	Sipho	Type-1_Cluster-6	no
Vibrio phage 1.038.O._10N.286.51.C2	*Vibrio lentus*	1537	49,567	43.1678	89	0	0	Myo	Type-1_Cluster-7	no
Vibrio phage 1.039.O._10N.286.55.A2	*Vibrio lentus*	11755	40,972	42.5242	68	0	0	Podo	Type-3	no
Vibrio phage 1.040.O._10N.286.45.B9	*Vibrio lentus*	3323	10,579	43.3217	21	0	1	.	.	no
Vibrio phage 1.042.O._10N.286.45.B8	*Vibrio lentus*	399	49,730	43.1751	95	0	0	Myo	Type-1_Cluster-7	no
Vibrio phage 1.043.O._10N.261.52.C7	*Vibrio lentus*	3772	10,272	41.8224	21	0	0	.	.	no
Vibrio phage 1.044.O._10N.261.51.B8	*Vibrio lentus*	3311	10,272	41.7932	21	0	0	.	.	no
Vibrio phage 1.046.O._10N.286.52.E3	*Vibrio splendidus*	6671	62,503	48.3225	89	2	2	Sipho	Type-1	no
Vibrio phage 1.047.O._10N.286.55.F2	*Vibrio cyclitrophicus*	3221	46,106	43.3002	73	0	1	Sipho	Type-1_Cluster-5	no
Vibrio phage 1.048.O._10N.286.46.A10	*Vibrio lentus*	2562	10,447	41.7249	22	0	0	.	.	no
Shewanella phage 1.049.O._10N.286.54.B5	*Shewanella sp.*	1142	45,021	41.5984	60	0	1	Sipho	Type-1_Cluster-6	no
Shewanella phage 1.050.O._10N.286.48.A6	*Shewanella sp.*	467	45,285	41.6407	60	0	1	Sipho	Type-1_Cluster-6	no
Enterovibrio phage 1.052.A._10N.286.46.C3	*Enterovibrio norvegicus*	453	42,889	46.7719	75	0	0	Myo	Type-1_Cluster-7	no
Vibrio phage 1.054.O._10N.261.52.A1	*Vibrio sp.*	771	41,774	42.3182	72	0	0	Myo	Type-1_Cluster-7	no
Enterovibrio phage 1.055.O._10N.286.55.E9	*Enterovibrio norvegicus*	461	47,199	40.9288	66	0	0	Sipho	Type-1_Cluster-6	no
Vibrio phage 1.056.O._10N.261.48.C11	*Vibrio lentus*	260	47,054	44.7571	53	0	0	Sipho	Type-1_Cluster-6	no
Vibrio phage 1.057.O._10N.261.46.B12	*Vibrio lentus*	3223	10,273	41.7794	21	0	0	.	.	no
Vibrio phage 1.060.A._10N.261.48.B5	*Vibrio splendidus*	528	41,105	43.2551	65	1	0	Sipho	Type-1_Cluster-5	no
Vibrio phage 1.061.O._10N.286.55.C2	*Vibrio lentus*	465	41,667	43.0749	65	0	0	Podo	Type-3	no
Vibrio phage 1.062.O._10N.286.55.C3	*Vibrio cyclitrophicus*	2774	10,579	43.3311	21	0	1	.	.	no
Vibrio phage 1.063.O._10N.261.45.C7	*Vibrio lentus*	166	128,641	37.3357	231	0	0	Myo	Type-1_Cluster-7	no
Vibrio phage 1.064.O._10N.261.52.E2	unknown	737	36,584	42.7892	61	0	0	Sipho	Type-1_Cluster-5	no
Vibrio phage 1.066.O._10N.286.46.E8	*Vibrio lentus*	412	47,053	44.7495	53	0	0	Sipho	Type-1_Cluster-6	no
Vibrio phage 1.067.O._10N.261.52.C9	*Vibrio lentus*	401	40,955	42.5418	78	0	0	Podo	Type-3	no
Vibrio phage 1.068.O._10N.261.51.F8	*Vibrio lentus*	997	47,038	44.7596	53	0	0	Sipho	Type-1_Cluster-6	no
Vibrio phage 1.069.O._10N.286.49.F11	*Vibrio lentus*	2906	10,579	43.3311	21	0	1	.	.	no
Enterovibrio phage 1.070.O._10N.261.45.B2	*Enterovibrio norvegicus*	709	44,087	49.3955	66	0	0	Podo	Type-3	no
Vibrio phage 1.071.A._10N.286.46.A12	*Vibrio lentus*	753	48,114	43.3096	87	0	0	Myo	Type-1_Cluster-7	no
Vibrio phage 1.072.O._10N.286.48.A12	unknown	95	43,788	44.7725	51	0	0	Sipho	Type-1_Cluster-6	no
Vibrio phage 1.074.O._10N.222.49.B7	*Vibrio breoganii*	205	36,922	42.6087	64	0	0	Sipho	Type-1_Cluster-5	no
Vibrio phage 1.075.O._10N.286.55.B10	*Vibrio sp. F12*	435	51,290	44.2893	73	0	0	Myo	Unassigned	no
Shewanella phage 1.076.O._10N.286.51.B7	*Shewanella sp.*	119	47,914	42.8434	73	1	0	Sipho	Type-1	no
Enterovibrio phage 1.077.O._10N.261.45.A10	*Enterovibrio norvegicus*	629	44,047	49.3791	66	0	0	Podo	Type-3	no
Vibrio phage 1.079.O._10N.286.45.E9	*Vibrio lentus*	606	42,375	42.7823	72	0	0	Podo	Type-3	no
Vibrio phage 1.080.O._10N.286.48.A4	*Vibrio lentus*	3663	10,046	41.1905	19	0	0	.	.	no
Shewanella phage 1.081.O._10N.286.52.C2	*Shewanella sp.*	88	239,318	42.4130	354	23	2	Myo	Type-2	no
Vibrio phage 1.082.O._10N.261.49.E4	*Vibrio breoganii*	275	35,810	42.9964	57	0	0	Sipho	Type-1_Cluster-5	no
Shewanella phage 1.083.O._10N.286.52.B9	*Shewanella sp.*	199	45,120	38.7079	61	0	2	Sipho	Type-1_Cluster-6	no
Enterovibrio phage 1.084.O._10N.261.49.F5	*Enterovibrio norvegicus*	43	141,906	37.1027	244	0	0	Myo	Unassigned	no
Vibrio phage 1.085.O._10N.222.51.E3	*Vibrio breoganii*	173	37,820	42.7393	62	2	0	Sipho	Type-1_Cluster-5	no
Vibrio phage 1.086.O._10N.222.51.F8	*Vibrio splendidus*	439	50,835	44.1920	68	0	0	Myo	Unassigned	no
Vibrio phage 1.087.A._10N.261.45.F9	*Vibrio lentus*	557	45,674	44.1192	67	0	0	Myo	Unassigned	no
Vibrio phage 1.088.O._10N.261.46.A1	*Vibrio lentus*	469	60,385	48.7820	90	1	0	Sipho	Type-1_Cluster-5	no
Vibrio phage 1.089.O._10N.261.51.F9	*Vibrio lentus*	116	59,851	48.5606	97	1	0	Sipho	Type-1_Cluster-5	no
Vibrio phage 1.090.B._10N.286.48.F1	*Vibrio lentus*	1032	41,868	42.5193	68	0	0	Podo	Type-3	no
Vibrio phage 1.091.O._10N.286.52.B12	*Vibrio lentus*	180	43,134	42.7667	75	0	0	Podo	Type-3	no
Vibrio phage 1.093.O._10N.286.55.E10	*Vibrio sp. F12*	287	51,290	44.2874	73	0	0	Myo	Unassigned	no
Vibrio phage 1.094.O._10N.286.55.E12	*Vibrio sp. F12*	296	51,290	44.2854	73	0	0	Myo	Unassigned	no
Vibrio phage 1.095.O._10N.286.46.E10	*Vibrio sp. F12*	3005	10,436	41.8168	22	0	0	.	.	no
Vibrio phage 1.097.O._10N.286.49.B3	*Vibrio sp.*	315	76,918	42.5960	98	0	0	Podo	Type-3	no
Vibrio phage 1.098.O._10N.286.51.B9	*Vibrio lentus*	138	59,851	48.5573	97	1	0	Sipho	Type-1_Cluster-5	no
Vibrio phage 1.100.O._10N.261.45.C3	*Vibrio lentus*	564	51,268	44.4059	70	0	0	Myo	Unassigned	no
Enterovibrio phage 1.101.O._10N.261.45.C6	*Enterovibrio norvegicus*	27	130,250	37.3589	224	2	0	Myo	Type-1_Cluster-7	no
Vibrio phage 1.102.O._10N.261.45.E3	*Vibrio lentus*	3460	10,447	41.7249	22	0	0	.	.	no
Vibrio phage 1.103.O._10N.261.52.F2	*Vibrio splendidus*	511	61,748	48.6348	93	1	0	Sipho	Type-1	no
Vibrio phage 1.104.O._10N.286.49.A12	*Vibrio lentus*	125	60,990	48.7506	93	0	0	Sipho	Type-1	no
Vibrio phage 1.105.O._10N.286.49.B4	*Vibrio cyclitrophicus*	4312	49,123	42.8089	80	0	0	Myo	Type-1_Cluster-7	no
Vibrio phage 1.106.O._10N.286.51.F7	*Vibrio cyclitrophicus*	174	47,070	42.8001	77	0	0	Myo	Type-1_Cluster-7	no
Vibrio phage 1.107.A._10N.286.52.E10	*Vibrio lentus*	4180	10,447	41.7058	22	0	0	.	.	no
Vibrio phage 1.107.B._10N.286.52.E10	*Vibrio lentus*	3583	10,447	41.7058	22	0	0	.	.	yes
Vibrio phage 1.107.C._10N.286.52.E10	*Vibrio lentus*	2984	10,447	41.7058	22	0	0	.	.	yes
Vibrio phage 1.108.O._10N.222.51.A4	*Vibrio breoganii*	168	36,584	42.7919	62	0	0	Sipho	Type-1_Cluster-5	no
Vibrio phage 1.110.O._10N.261.52.C1	*Vibrio breoganii*	224	37,556	42.9226	66	1	0	Sipho	Type-1_Cluster-5	no
Vibrio phage 1.111.A._10N.286.45.E6	*Vibrio lentus*	730	40,209	43.5748	62	0	0	Myo	Type-1_Cluster-6	no
Vibrio phage 1.111.B._10N.286.45.E6	*Vibrio lentus*	795	40,209	43.5748	62	0	0	Myo	Type-1_Cluster-6	yes
Vibrio phage 1.112.O._10N.286.46.B11	*Vibrio lentus*	643	48,149	43.0829	83	0	0	Myo	Type-1_Cluster-7	no
Vibrio phage 1.113.A._10N.286.51.E7	*Vibrio splendidus*	547	44,150	43.3431	74	0	0	Sipho	Type-1_Cluster-5	no
Vibrio phage 1.115.A._10N.222.49.B11	*Vibrio breoganii*	261	37,416	43.2248	71	0	0	Sipho	Type-1_Cluster-5	no
Vibrio phage 1.115.B._10N.222.49.B11	*Vibrio breoganii*	822	37,416	43.2222	71	0	0	Sipho	Type-1_Cluster-5	yes
Vibrio phage 1.116.O._10N.222.52.C10	*Vibrio breoganii*	245	36,314	42.6833	61	2	0	Sipho	Type-1_Cluster-5	no
Vibrio phage 1.117.O._10N.261.45.E9	*Vibrio breoganii*	459	55,794	49.4713	82	0	0	Sipho	Type-1_Cluster-5	no
Vibrio phage 1.118.A._10N.261.49.F6	*Vibrio lentus*	308	60,458	48.7959	96	1	0	Sipho	Type-1_Cluster-5	no
Vibrio phage 1.118.B._10N.261.49.F6	*Vibrio lentus*	357	60,458	48.7959	96	1	0	Sipho	Type-1_Cluster-5	yes
Vibrio phage 1.119.O._10N.261.51.A9	*Vibrio lentus*	166	44,527	42.1497	76	0	0	Sipho	Type-1_Cluster-5	no
Enterovibrio phage 1.121.O._10N.286.46.C4	*Enterovibrio norvegicus*	36	145,590	39.6861	263	3	0	Myo	Type-1_Cluster-7	no
Vibrio phage 1.122.A._10N.286.46.F8	*Vibrio cyclitrophicus*	418	44,523	43.6246	69	2	0	Sipho	Type-1	no
Vibrio phage 1.122.B._10N.286.46.F8	*Vibrio cyclitrophicus*	450	44,523	43.6291	69	2	0	Sipho	Type-1	yes
Enterovibrio phage 1.123.O._10N.286.48.F3	*Enterovibrio norvegicus*	186	46,071	48.2972	72	0	0	Myo	Type-1_Cluster-7	no
Vibrio phage 1.124.O._10N.286.49.B1	*Vibrio sp.*	220	47,604	43.5216	70	0	1	Myo	Unassigned	no
Vibrio phage 1.125.O._10N.286.49.F5	*Vibrio splendidus*	4584	10,578	43.3541	21	0	1	.	.	no
Vibrio phage 1.127.O._10N.286.52.E12	*Vibrio lentus*	169	44,915	42.2977	77	0	0	Sipho	Type-1_Cluster-5	no
Vibrio phage 1.131.O._10N.222.49.A8	*Vibrio breoganii*	197	37,933	42.8387	67	0	0	Sipho	Type-1_Cluster-5	no
Vibrio phage 1.132.O._10N.222.49.F8	*Vibrio breoganii*	187	36,926	43.2053	65	0	0	Sipho	Type-1_Cluster-5	no
Vibrio phage 1.133.O._10N.222.51.E4	*Vibrio breoganii*	132	37,087	42.8991	66	0	0	Sipho	Type-1_Cluster-5	no
Vibrio phage 1.134.O._10N.222.52.B8	*Vibrio breoganii*	144	36,379	42.9561	62	2	0	Sipho	Type-1_Cluster-5	no
Vibrio phage 1.135.O._10N.222.54.B6	*Vibrio sp. F13*	411	41,918	42.5450	75	0	0	Sipho	Type-1_Cluster-5	no
Vibrio phage 1.136.O._10N.261.45.E11	*Vibrio sp.*	206	31,617	45.9436	50	0	0	Myo	Type-1_Cluster-8	no
Vibrio phage 1.137.O._10N.261.46.B5	*Vibrio lentus*	523	37,958	42.4100	69	0	0	Sipho	Type-1_Cluster-5	no
Vibrio phage 1.138.O._10N.261.48.A1	*Vibrio lentus*	527	32,510	45.7982	45	0	0	Podo	Type-3	no
Vibrio phage 1.139.A._10N.261.48.C6	*Vibrio breoganii*	96	43,893	47.9553	67	0	1	Podo	Type-3	no
Vibrio phage 1.139.B._10N.261.48.C6	*Vibrio breoganii*	488	44,094	48.0224	68	0	1	Podo	Type-3	yes
Vibrio phage 1.141.A._10N.261.49.B3	*Vibrio kanaloae*	6889	10,047	41.1566	19	0	0	.	.	no
Vibrio phage 1.142.O._10N.261.49.E11	*Vibrio sp.*	732	31,617	45.9436	50	0	0	Myo	Type-1_Cluster-8	no
Vibrio phage 1.143.O._10N.261.55.C8	*Vibrio lentus*	194	44,527	42.1475	76	0	0	Sipho	Type-1_Cluster-5	no
Vibrio phage 1.144.O._10N.286.45.B3	*Vibrio splendidus*	153	44,418	42.6336	75	0	0	Sipho	Type-1_Cluster-5	no
Vibrio phage 1.147.O._10N.286.49.E9	*Vibrio breoganii*	165	37,360	42.8132	66	0	0	Sipho	Type-1_Cluster-5	no
Vibrio phage 1.148.O._10N.286.54.A10	*Vibrio breoganii*	537	36,789	42.4475	65	0	0	Sipho	Type-1_Cluster-5	no
Vibrio phage 1.149.O._10N.286.55.A12	*Vibrio cyclitrophicus*	163	48,481	42.7466	79	0	0	Myo	Type-1_Cluster-7	no
Vibrio phage 1.150.O._10N.222.46.A6	*Vibrio splendidus*	127	75,796	43.1065	108	1	0	Podo	Type-3	no
Vibrio phage 1.151.O._10N.222.46.B1	*Vibrio splendidus*	225	44,307	44.3745	64	0	0	Sipho	Type-1_Cluster-5	no
Vibrio phage 1.152.O._10N.222.46.E1	*Vibrio splendidus*	237	75,798	43.1040	108	1	0	Podo	Type-3	no
Vibrio phage 1.154.O._10N.222.52.B12	*Vibrio sp.*	511	37,136	42.2070	60	0	1	Myo	Type-1_Cluster-7	no
Vibrio phage 1.155.O._10N.222.55.B3	*Vibrio sp. F13*	1070	29,029	44.0008	45	0	0	Myo	Unassigned	no
Vibrio phage 1.156.O._10N.261.45.A6	*Vibrio sp.*	301	31,617	45.9436	50	0	0	Myo	Type-1_Cluster-8	no
Vibrio phage 1.157.O._10N.261.45.B7	*Vibrio breoganii*	206	35,855	42.9201	59	0	0	Sipho	Type-1_Cluster-5	no
Vibrio phage 1.158.O._10N.261.45.E12	*Vibrio lentus*	110	46,507	44.6191	72	0	0	Myo	Unassigned	no
Vibrio phage 1.159.O._10N.261.46.F12	*Vibrio sp.*	997	31,617	45.9468	50	0	0	Myo	Type-1_Cluster-8	no
Vibrio phage 1.160.O._10N.261.48.B11	*Vibrio lentus*	201	60,734	48.6548	93	1	0	Sipho	Type-1_Cluster-5	no
Vibrio phage 1.161.O._10N.261.48.C5	*Vibrio lentus*	71	140,668	37.6802	228	0	1	Myo	Type-1_Cluster-7	no
Vibrio phage 1.162.O._10N.261.48.E3	*Vibrio breoganii*	216	37,360	42.8105	66	0	0	Sipho	Type-1_Cluster-5	no
Vibrio phage 1.164.O._10N.261.51.A7	*Vibrio sp. F13*	677	48,235	44.0282	64	0	1	Myo	Unassigned	no
Vibrio phage 1.165.O._10N.261.51.B7	*Vibrio breoganii*	861	37,046	42.9898	61	0	0	Sipho	Type-1_Cluster-5	no
Vibrio phage 1.166.O._10N.261.51.C7	*Vibrio lentus*	214	21,800	45.9679	35	0	0	Myo	Unassigned	no
Vibrio phage 1.167.O._10N.261.51.F2	*Vibrio breoganii*	430	35,811	43.0482	66	0	0	Sipho	Type-1_Cluster-5	no
Vibrio phage 1.168.O._10N.261.52.A10	*Vibrio breoganii*	154	37,551	42.5555	66	0	0	Sipho	Type-1_Cluster-5	no
Vibrio phage 1.169.O._10N.261.52.B1	*Vibrio breoganii*	139	72,290	42.9409	85	1	0	Podo	Type-3	no
Vibrio phage 1.170.O._10N.261.52.C3	*Vibrio sp.*	65	133,692	38.6964	214	0	0	Myo	Type-1_Cluster-7	no
Vibrio phage 1.171.O._10N.261.52.F12	*Vibrio lentus*	71	60,216	48.7246	88	0	0	Sipho	Type-1	no
Vibrio phage 1.172.O._10N.261.52.F5	*Vibrio breoganii*	262	36,314	42.6833	61	2	0	Sipho	Type-1_Cluster-5	no
Vibrio phage 1.173.O._10N.261.55.A11	*Vibrio cyclitrophicus*	81	48,063	42.7585	84	0	0	Myo	Type-1_Cluster-7	no
Vibrio phage 1.174.O._10N.261.55.A8	*Vibrio lentus*	145	47,063	43.9496	68	0	0	Myo	Unassigned	no
Vibrio phage 1.175.O._10N.261.55.B3	*Vibrio lentus*	593	49,066	44.5135	74	0	0	Myo	Unassigned	no
Vibrio phage 1.176.O._10N.261.55.F5	*Vibrio breoganii*	123	36,463	42.9339	63	2	0	Sipho	Type-1_Cluster-5	no
Vibrio phage 1.177.O._10N.286.45.E10	*Vibrio sp. F12*	372	45,439	42.1158	80	0	0	Sipho	Type-1_Cluster-5	no
Vibrio phage 1.178.O._10N.286.45.E12	*Vibrio cyclitrophicus*	449	39,934	41.8491	74	0	0	Sipho	Type-1_Cluster-5	no
Vibrio phage 1.179.O._10N.286.45.F12	*Vibrio splendidus*	1499	31,498	45.6823	45	0	0	Podo	Type-3	no
Vibrio phage 1.181.O._10N.286.46.C9	*Vibrio splendidus*	122	50,228	43.3782	78	0	0	Myo	Unassigned	no
Vibrio phage 1.182.O._10N.286.46.E1	*Vibrio breoganii*	829	36,910	42.7283	56	0	0	Podo	Type-3	no
Vibrio phage 1.183.O._10N.286.48.B7	*Vibrio sp.*	91	37,411	44.0165	41	0	1	Podo	Type-3	no
Vibrio phage 1.184.A._10N.286.49.A5	*Vibrio cyclitrophicus*	862	33,272	43.7936	48	0	1	Podo	Type-3	no
Vibrio phage 1.185.O._10N.286.49.C2	*Vibrio sp.*	484	43,397	46.0447	48	0	1	Podo	Type-3	no
Vibrio phage 1.186.O._10N.286.49.E3	*Vibrio cyclitrophicus*	195	48,643	42.7831	78	2	0	Myo	Type-1_Cluster-7	no
Vibrio phage 1.187.O._10N.286.49.F1	*Vibrio splendidus*	370	133,254	37.9268	255	2	0	Myo	Type-1_Cluster-7	no
Vibrio phage 1.188.A._10N.286.51.A6	*Vibrio breoganii*	99	72,305	42.9832	86	1	0	Podo	Type-3	no
Vibrio phage 1.188.B._10N.286.51.A6	*Vibrio breoganii*	300	72,305	42.9832	86	1	0	Podo	Type-3	yes
Vibrio phage 1.188.C._10N.286.51.A6	*Vibrio breoganii*	302	72,305	42.9832	86	1	0	Podo	Type-3	yes
Vibrio phage 1.189.B._10N.286.51.B5	*Vibrio breoganii*	720	36,855	42.7974	66	0	0	Sipho	Type-1_Cluster-5	yes
Vibrio phage 1.189.C._10N.286.51.B5	*Vibrio breoganii*	719	36,855	42.8002	66	0	0	Sipho	Type-1_Cluster-5	yes
Vibrio phage 1.189.O._10N.286.51.B5	*Vibrio breoganii*	469	36,855	42.7974	66	0	0	Sipho	Type-1_Cluster-5	no
Vibrio phage 1.190.O._10N.286.51.F12	*Vibrio lentus*	1810	21,785	45.9353	35	0	0	Myo	Unassigned	no
Vibrio phage 1.191.O._10N.286.52.B4	*Vibrio splendidus*	118	48,354	44.1970	72	0	0	Myo	Unassigned	no
Vibrio phage 1.193.O._10N.286.52.C6	*Vibrio splendidus*	207	132,560	38.0854	249	4	0	Myo	Type-1_Cluster-7	no
Vibrio phage 1.194.O._10N.286.54.B1	*Vibrio lentus*	110	59,294	48.5800	85	0	0	Sipho	Type-1	no
Vibrio phage 1.195.O._10N.286.54.C8	*Vibrio lentus*	108	59,738	48.6524	88	0	0	Sipho	Type-1	no
Vibrio phage 1.196.O._10N.286.54.E12	*Vibrio lentus*	146	59,414	48.5795	85	0	0	Sipho	Type-1	no
Vibrio phage 1.197.A._10N.286.54.F2	*Vibrio cyclitrophicus*	224	44,587	43.6181	71	0	0	Sipho	Type-1_Cluster-5	no
Vibrio phage 1.198.A._10N.286.54.F4	*Vibrio cyclitrophicus*	576	44,472	41.9050	79	0	0	Sipho	Type-1_Cluster-5	no
Vibrio phage 1.198.B._10N.286.54.F4	*Vibrio cyclitrophicus*	1334	44,343	41.8578	78	0	0	Sipho	Type-1_Cluster-5	yes
Vibrio phage 1.199.A._10N.286.55.C10	*Vibrio cyclitrophicus*	803	48,312	43.0452	77	0	0	Myo	Type-1_Cluster-7	no
Vibrio phage 1.199.B._10N.286.55.C10	*Vibrio cyclitrophicus*	851	48,312	43.0431	77	0	0	Myo	Type-1_Cluster-7	yes
Vibrio phage 1.200.O._10N.286.55.E1	*Vibrio lentus*	513	59,297	48.5927	85	0	0	Sipho	Type-1	no
Vibrio phage 1.201.B._10N.286.55.F1	*Vibrio cyclitrophicus*	730	50,506	44.2918	73	0	0	Myo	Unassigned	no
Vibrio phage 1.202.O._10N.222.45.E8	*Vibrio cyclitrophicus*	1174	32,014	44.5586	42	0	0	Myo	Type-1_Cluster-9	no
Vibrio phage 1.204.O._10N.222.46.F12	*Vibrio cyclitrophicus*	658	44,168	43.5157	55	0	0	Podo	Type-3	no
Vibrio phage 1.205.O._10N.222.51.A7	*Vibrio tasmaniensis*	167	57,861	44.9249	70	0	0	Podo	Type-3	no
Vibrio phage 1.206.O._10N.222.51.B10	*Vibrio breoganii*	744	37,551	42.9256	62	0	0	Sipho	Type-1_Cluster-5	no
Vibrio phage 1.207.B._10N.222.51.C2	*Vibrio breoganii*	252	55,793	49.4757	81	0	0	Sipho	Type-1_Cluster-5	no
Vibrio phage 1.208.B._10N.222.52.A7	*Vibrio splendidus*	517	48,927	44.4642	69	2	0	Podo	Type-3	no
Vibrio phage 1.209.O._10N.222.52.B2	*Vibrio breoganii*	508	43,712	48.0005	65	0	1	Podo	Type-3	no
Vibrio phage 1.210.O._10N.222.52.C2	*Vibrio sp. F13*	117	48,224	42.0392	74	1	0	Sipho	Type-1	no
Vibrio phage 1.211.A._10N.222.52.F11	*Vibrio tasmaniensis*	736	37,169	43.7488	40	0	0	Podo	Type-3	no
Vibrio phage 1.211.B._10N.222.52.F11	*Vibrio tasmaniensis*	119	37,169	43.7488	40	0	0	Podo	Type-3	yes
Vibrio phage 1.213.O._10N.222.54.F10	*Vibrio sp. F13*	208	42,443	42.5677	73	0	0	Sipho	Type-1_Cluster-5	no
Vibrio phage 1.214.O._10N.222.54.F11	*Vibrio cyclitrophicus*	173	44,205	43.6285	69	1	0	Sipho	Type-1	no
Vibrio phage 1.215.A._10N.222.54.F7	*Vibrio splendidus*	118	80,834	45.5477	105	0	0	Sipho	Type-1_Cluster-9	no
Vibrio phage 1.215.B._10N.222.54.F7	*Vibrio splendidus*	65	80,834	45.5477	105	0	0	Sipho	Type-1_Cluster-9	yes
Vibrio phage 1.216.O._10N.222.55.C12	*Vibrio sp. F13*	235	41,359	42.6026	69	0	0	Sipho	Type-1_Cluster-5	no
Vibrio phage 1.217.O._10N.261.45.A1	*Vibrio sp.*	334	31,617	45.9436	50	0	0	Myo	Type-1_Cluster-8	no
Vibrio phage 1.219.O._10N.261.45.E2	*Vibrio sp.*	710	31,617	45.9436	50	0	0	Myo	Type-1_Cluster-8	no
Vibrio phage 1.223.O._10N.261.48.A9	*Vibrio lentus*	593	49,535	44.4433	70	0	0	Myo	Unassigned	no
Vibrio phage 1.224.A._10N.261.48.B1	*Vibrio breoganii*	95	71,915	43.0633	85	1	0	Podo	Type-3	no
Vibrio phage 1.225.O._10N.261.48.B7	*Vibrio splendidus*	358	52,615	44.2421	75	0	0	Myo	Unassigned	no
Vibrio phage 1.226.O._10N.261.48.E5	*Vibrio breoganii*	256	35,655	42.9785	67	0	0	Sipho	Type-1_Cluster-5	no
Vibrio phage 1.228.O._10N.261.49.C1	*Vibrio breoganii*	255	36,667	42.8969	64	0	0	Sipho	Type-1_Cluster-5	no
Vibrio phage 1.231.O._10N.261.49.F8	*Vibrio lentus*	1131	44,527	42.1430	76	0	0	Sipho	Type-1_Cluster-5	no
Vibrio phage 1.232.O._10N.261.51.E11	*Vibrio tasmaniensis*	92	56,736	43.8963	79	0	0	Podo	Type-3	no
Vibrio phage 1.233.A._10N.261.51.E6	*Vibrio breoganii*	789	36,823	43.1144	60	0	0	Sipho	Type-1_Cluster-5	no
Vibrio phage 1.233.B._10N.261.51.E6	*Vibrio breoganii*	657	36,823	43.1144	60	0	0	Sipho	Type-1_Cluster-5	yes
Vibrio phage 1.235.O._10N.261.52.B2	*Vibrio lentus*	2261	47,017	43.0249	59	0	0	Podo	Type-3	no
Vibrio phage 1.236.O._10N.261.52.C4	*Vibrio cyclitrophicus*	482	42,646	42.3252	67	0	0	Sipho	Type-1_Cluster-5	no
Vibrio phage 1.237.A._10N.261.52.C5	*Vibrio lentus*	80	60,097	48.7628	95	1	0	Sipho	Type-1_Cluster-5	no
Vibrio phage 1.237.B._10N.261.52.C5	*Vibrio lentus*	377	60,160	48.7633	95	1	0	Sipho	Type-1_Cluster-5	yes
Vibrio phage 1.238.A._10N.261.52.F10	*Vibrio breoganii*	485	70,494	43.1881	93	0	0	Podo	Type-3	no
Vibrio phage 1.238.B._10N.261.52.F10	*Vibrio breoganii*	86	70,467	43.1890	94	0	0	Podo	Type-3	yes
Vibrio phage 1.239.O._10N.261.52.F6	*Vibrio breoganii*	249	38,618	43.0214	64	0	1	Sipho	Type-1_Cluster-5	no
Vibrio phage 1.240.O._10N.261.52.F8	*Vibrio breoganii*	511	36,710	42.5306	59	1	0	Sipho	Type-1_Cluster-5	no
Vibrio phage 1.242.O._10N.261.54.B2	*Vibrio breoganii*	212	36,910	42.7526	64	1	0	Sipho	Type-1_Cluster-5	no
Vibrio phage 1.243.O._10N.261.54.B5	*Vibrio cyclitrophicus*	163	48,414	42.9401	79	2	0	Myo	Type-1_Cluster-7	no
Vibrio phage 1.244.A._10N.261.54.C3	*Vibrio sp. F13*	219	159,885	44.3350	211	3	1	Myo	Type-2	no
Vibrio phage 1.245.O._10N.261.54.C7	*Vibrio breoganii*	303	71,702	43.1201	94	0	0	Podo	Type-3	no
Vibrio phage 1.246.O._10N.261.54.E10	*Vibrio sp. F13*	361	47,133	44.7606	67	0	0	Myo	Unassigned	no
Vibrio phage 1.247.A._10N.261.54.E12	*Vibrio splendidus*	536	43,896	44.0564	70	1	0	Sipho	Type-1	no
Vibrio phage 1.247.B._10N.261.54.E12	*Vibrio splendidus*	673	43,896	44.0541	70	1	0	Sipho	Type-1	yes
Vibrio phage 1.248.O._10N.261.54.F1	*Vibrio cyclitrophicus*	3805	48,301	42.8811	80	0	0	Myo	Type-1_Cluster-7	no
Vibrio phage 1.249.A._10N.261.55.B9	*Vibrio cyclitrophicus*	3423	10,611	46.5555	22	0	0	.	.	no
Vibrio phage 1.249.B._10N.261.55.B9	*Vibrio cyclitrophicus*	2160	10,611	46.5555	22	0	0	.	.	yes
Vibrio phage 1.250.O._10N.261.55.E11	*Vibrio splendidus*	253	59,981	48.9305	94	1	0	Sipho	Type-1	no
Vibrio phage 1.251.O._10N.261.55.E5	*Vibrio lentus*	298	59,649	48.5658	93	1	0	Sipho	Type-1	no
Vibrio phage 1.253.O._10N.286.45.B12	*Vibrio cyclitrophicus*	611	47,008	43.0182	59	0	0	Podo	Type-3	no
Vibrio phage 1.254.O._10N.286.45.C8	*Vibrio lentus*	676	32,699	45.4662	45	0	0	Podo	Type-3	no
Vibrio phage 1.255.O._10N.286.45.F1	*Vibrio splendidus*	198	159,885	44.3350	211	3	1	Myo	Type-2	no
Vibrio phage 1.256.O._10N.286.45.F8	*Vibrio cyclitrophicus*	123	48,207	42.9668	76	0	0	Myo	Type-1_Cluster-7	no
Vibrio phage 1.257.O._10N.286.46.A4	*Vibrio splendidus*	774	32,371	45.7755	47	0	0	Podo	Type-3	no
Vibrio phage 1.259.O._10N.286.48.F4	*Vibrio splendidus*	371	28,145	43.7733	42	0	0	Myo	Unassigned	no
Vibrio phage 1.261.O._10N.286.51.A7	*Vibrio breoganii*	376	71,992	43.1659	82	1	0	Podo	Type-3	no
Vibrio phage 1.262.O._10N.286.51.A9	*Vibrio breoganii*	325	47,635	39.0721	74	4	0	Podo	Type-3	no
Vibrio phage 1.263.A._10N.286.51.B1	*Vibrio cyclitrophicus*	756	49,640	43.0379	76	0	0	Myo	Type-1_Cluster-7	no
Vibrio phage 1.263.B._10N.286.51.B1	*Vibrio cyclitrophicus*	64	49,640	43.0419	76	0	0	Myo	Type-1_Cluster-7	yes
Vibrio phage 1.264.O._10N.286.51.F2	*Vibrio splendidus*	270	47,739	42.7701	67	0	0	Podo	Type-3	no
Vibrio phage 1.265.O._10N.286.52.F6	*Vibrio cyclitrophicus*	405	43,630	42.1797	70	0	0	Sipho	Type-1_Cluster-5	no
Vibrio phage 1.266.O._10N.286.52.F9	*Vibrio breoganii*	257	34,788	43.2620	59	0	0	Sipho	Type-1_Cluster-5	no
Vibrio phage 1.267.O._10N.286.54.A1	*Vibrio lentus*	95	59,414	48.5795	85	0	0	Sipho	Type-1	no
Vibrio phage 1.268.A._10N.286.54.A11	*Vibrio lentus*	435	59,297	48.5876	85	0	0	Sipho	Type-1	no
Vibrio phage 1.268.B._10N.286.54.A11	*Vibrio lentus*	342	59,297	48.5876	85	0	0	Sipho	Type-1	yes
Vibrio phage 1.269.O._10N.286.54.A6	*Vibrio lentus*	85	59,738	48.6524	88	0	0	Sipho	Type-1	no
Vibrio phage 1.270.A._10N.286.54.A8	*Vibrio lentus*	71	59,294	48.5918	85	0	0	Sipho	Type-1	no
Vibrio phage 1.270.B._10N.286.54.A8	*Vibrio lentus*	44	59,294	48.5901	85	0	0	Sipho	Type-1	yes
Vibrio phage 1.271.A._10N.286.54.B4	*Vibrio lentus*	170	59,297	48.5859	85	0	0	Sipho	Type-1	no
Vibrio phage 1.271.B._10N.286.54.B4	*Vibrio lentus*	411	59,297	48.5893	85	0	0	Sipho	Type-1	yes
Vibrio phage 1.272.O._10N.286.54.C4	*Vibrio lentus*	286	59,297	48.5910	85	0	0	Sipho	Type-1	no
Vibrio phage 1.273.O._10N.286.54.C7	*Vibrio cyclitrophicus*	159	48,145	42.8601	78	2	0	Myo	Type-1_Cluster-7	no
Vibrio phage 1.274.O._10N.286.54.E1	*Vibrio lentus*	159	59,409	48.5718	85	0	0	Sipho	Type-1	no
Vibrio phage 1.275.O._10N.286.54.E11	*Vibrio cyclitrophicus*	256	37,915	44.3044	53	1	0	Podo	Type-3	no
Vibrio phage 1.276.O._10N.286.54.E4	*Vibrio cyclitrophicus*	130	44,334	42.1843	72	0	0	Sipho	Type-1_Cluster-5	no
Vibrio phage 1.277.A._10N.286.54.E7	*Vibrio lentus*	337	59,297	48.5876	85	0	0	Sipho	Type-1	no
Vibrio phage 1.277.B._10N.286.54.E7	*Vibrio lentus*	482	59,419	48.6023	85	0	0	Sipho	Type-1	yes
Vibrio phage 1.278.O._10N.286.54.E8	*Vibrio cyclitrophicus*	387	49,282	42.6687	82	0	0	Myo	Type-1_Cluster-7	no
Vibrio phage 1.280.O._10N.286.54.F6	*Vibrio lentus*	373	59,297	48.5910	85	0	0	Sipho	Type-1	no
Vibrio phage 1.281.O._10N.286.54.F7	*Vibrio lentus*	78	59,297	48.5910	85	0	0	Sipho	Type-1	no
Vibrio phage 1.282.A._10N.286.54.F8	*Vibrio lentus*	255	59,359	48.5773	85	0	0	Sipho	Type-1	no
Vibrio phage 1.283.A._10N.286.55.A1	*Vibrio lentus*	532	59,530	48.6209	85	0	0	Sipho	Type-1	no
Vibrio phage 1.283.B._10N.286.55.A1	*Vibrio lentus*	427	59,530	48.6209	85	0	0	Sipho	Type-1	yes
Vibrio phage 1.283.C._10N.286.55.A1	*Vibrio lentus*	370	59,530	48.6192	85	0	0	Sipho	Type-1	yes
Vibrio phage 1.284.A._10N.286.55.A5	*Vibrio cyclitrophicus*	663	45,648	43.4543	71	0	0	Sipho	Type-1_Cluster-5	no
Vibrio phage 1.285.O._10N.286.55.C12	*Vibrio lentus*	809	59,437	48.5522	93	1	0	Sipho	Type-1	no
Vibrio phage 1.286.O._10N.286.55.C4	*Vibrio cyclitrophicus*	575	49,131	42.9932	82	0	0	Myo	Type-1_Cluster-7	no
Vibrio phage 1.287.O._10N.286.55.C7	*Vibrio cyclitrophicus*	720	45,562	43.3344	71	0	0	Sipho	Type-1_Cluster-5	no
Vibrio phage 1.289.A._10N.286.55.E8	*Vibrio cyclitrophicus*	1521	44,529	43.3919	70	0	0	Sipho	Type-1_Cluster-5	no
Vibrio phage 1.291.O._10N.286.55.F6	*Vibrio cyclitrophicus*	759	43,662	42.9687	74	1	0	Sipho	Type-1_Cluster-5	no
Vibrio phage 1.293.O._10N.261.52.E1	*Vibrio cyclitrophicus*	413	48,275	44.1409	76	1	0	Podo	Type-3	no
Vibrio phage 2.044.O._10N.261.51.B8	*Vibrio lentus*	733	45,020	44.8156	53	0	2	Sipho	Type-1_Cluster-6	no
Vibrio phage 2.058.O._10N.286.46.B8	*Vibrio lentus*	322	101,637	41.0648	143	0	0	Sipho	Type-1_Cluster-6	no
Vibrio phage 2.092.O._10N.286.52.B7	*Vibrio lentus*	2518	10,580	43.3176	21	0	1	.	.	no
Vibrio phage 2.095.A._10N.286.46.E10	*Vibrio sp. F12*	578	44,649	42.3638	77	0	0	Sipho	Type-1_Cluster-5	no
Vibrio phage 2.095.B._10N.286.46.E10	*Vibrio sp. F12*	607	44,649	42.3593	77	0	0	Sipho	Type-1_Cluster-5	yes
Shewanella phage 2.096.O._10N.286.48.B5	*Shewanella sp.*	611	44,683	38.7843	57	0	2	Sipho	Type-1_Cluster-6	no
Vibrio phage 2.117.O._10N.261.45.E9	*Vibrio breoganii*	438	55,795	49.4704	82	0	0	Sipho	Type-1_Cluster-5	no
Vibrio phage 2.130.O._10N.222.46.C2	*Vibrio splendidus*	411	75,797	43.1099	107	1	0	Podo	Type-3	no
Vibrio phage 2.159.A._10N.261.46.F12	*Vibrio sp.*	1185	31,617	45.9436	50	0	0	Myo	Type-1_Cluster-8	no
Vibrio phage 2.159.B._10N.261.46.F12	*Vibrio sp.*	922	31,617	45.9436	50	0	0	Myo	Type-1_Cluster-8	yes
Vibrio phage 2.275.O._10N.286.54.E11	*Vibrio cyclitrophicus*	51	348,911	38.0928	489	19	1	Myo	Type-2	no
Vibrio phage 3.058.O._10N.286.46.B8	*Vibrio lentus*	295	101,642	41.0677	143	0	0	Sipho	Type-1_Cluster-6	no

**Table 2:** Strain identifier nomenclature, using example virus 1.008.O_10N.286.54.E5 and host 10N.286.54.E5.

Name component	Specific Description
1	A unique identifier for each independent plaque isolated from a given host from the initial exposure of a given host to an environmental virus concentrate.
008	A unique working ID for a host strain.
O	A lineage generated from a single plaque during viral serial purification, for example due to the emergence of multiple plaque morphologies. Options: Single lineage: O; Sub-lineages: A, B, C, etc….
10N	Year & site identifier (2010, Nahant).
286	Ordinal day.
54	A code representing the size-fraction of origin. Options: 0.2um: 45,46,47; 1um: 48,49,50; 5um: 51,52,53; 63um: 54,55,56. Note: Multiple codes within the size-fraction identifier reflect independent water samples for the 63um fraction, and independent water sample fractionation series for the other size classes (water sample A: 45,51,54; sample B: 46, 52, 55; sample C: 47, 53, 56).
E5	Unique storage well identifier.

**Table 3:** Replicate virus genome comparisons.

Phage	Query	Query Length	Subject	Subject Length	Query Mismatch (bp)	Subject Mismatch (bp)	ANI	SNP (bp)	inDEL (bp)	Start Variation (bp)	Length Variation (bp)	MAFFT Alignment # of diffs
**1.021**	A	43,743	B	43,743	1	1	0.9999771	0	0	0	0	0
**1.021**	A	43,743	C	43,743	2	2	0.9999543	1	0	0	0	0
**1.021**	B	43,743	C	43,743	2	2	0.9999543	0	0	0	0	0
**1.107**	B	10,447	C	10,447	1	0	0.9999521	0	0	0	0	0
**1.107**	B	10,447	A	10,447	3	2	0.9997607	2	0	0	0	2
**1.107**	C	10,447	A	10,447	3	2	0.9997607	2	0	0	0	2
**1.111**	A	40,209	B	40,209	10	9	0.9997637	0	0	9	0	0
**1.115**	B	37,416	A	37,416	2	3	0.9999332	1	0	1	0	0
**1.118**	B	60,458	A	60,458	5	5	0.9999173	4	0	0	0	4
**1.122**	A	44,523	B	44,523	3	4	0.9999214	2	0	1	0	2
**1.139**	B	44,094	A	43,893	6	1	0.9999204	0	201	48	201	201
**1.188**	A	72,305	B	72,305	2	3	0.9999654	0	0	2	0	0
**1.188**	A	72,305	C	72,305	2	3	0.9999654	0	0	2	0	0
**1.188**	B	72,305	C	72,305	1	1	0.9999862	0	0	0	0	0
**1.189**	B	36,855	O	36,855	4	3	0.9999050	2	0	1	0	2
**1.189**	B	36,855	C	36,855	4	4	0.9998915	3	0	0	0	3
**1.189**	O	36,855	C	36,855	2	3	0.9999322	1	0	1	0	1
**1.198**	B	44,343	A	44,472	2	43	0.9994933	1	129	0	129	130
**1.199**	B	48,312	A	48,312	4	4	0.9999172	3	0	0	0	3
**1.211**	A	37,169	B	37,169	1	1	0.9999731	0	0	0	0	0
**1.215**	B	80,834	A	80,834	1	2	0.9999814	0	0	1	0	0
**1.233**	B	36,823	A	36,823	2	1	0.9999593	0	0	1	0	0
**1.237**	B	60,160	A	60,097	64	3	0.9994429	0	63	1	63	64
**1.238**	A	70,467	B	70,494	19	20	0.9997233	0	27	19	27	27
**1.247**	B	43,896	A	43,896	3	2	0.9999430	1	0	1	0	1
**1.249**	B	10,611	A	10,611	1	0	0.9999529	0	0	0	0	0
**1.263**	B	49,640	A	49,640	11	12	0.9997683	2	0	9	0	0
**1.268**	A	59,297	B	59,297	3	3	0.9999494	2	0	0	0	2
**1.270**	B	59,297	A	59,297	3	2	0.9999578	1	0	1	0	1
**1.271**	B	59,297	A	59,297	3	3	0.9999494	2	0	0	0	2
**1.277**	A	59,419	B	59,297	3	21	0.9997978	1	122	1	122	123
**1.283**	A	59,530	C	59,530	3	2	0.9999580	1	0	0	0	1
**1.283**	A	59,530	B	59,530	1	1	0.9999832	0	0	0	0	0
**1.283**	C	59,530	B	59,530	2	3	0.9999580	1	0	0	0	1
**2.095**	B	44,649	A	44,649	3	4	0.9999216	2	0	1	0	2
**2.159**	A	31,617	B	31,617	0	0	1.0000000	0	0	0	0	0

## References

[d1] GenBank 2016 MG592390-MG592672

[d2] figshare Kauffman K. M. *et al.* 2018 https://dx.doi.org/10.6084/m9.figshare.c.4028239

