# Supplementary material for: Viruses of the Nahant Collection, characterization of 251 marine Vibrionaceae viruses
Source: Sci Data. 2018 Jul 3;5:180114. doi: 10.1038/sdata.2018.114 (PMC6029569; doi:10.1038/sdata.2018.114)
Supplement: Supplementary Information [file sdata2018114-s2.pdf]

Supplementary Figures - Table of Contents

Supplementary Figure 1……………………………………………………………………..…2
Supplementary Figure 2……………………………………………………………………….3
Supplementary Figure 3……………………………………………………………………….4
Supplementary Figure 4……………………………………………………………………….5

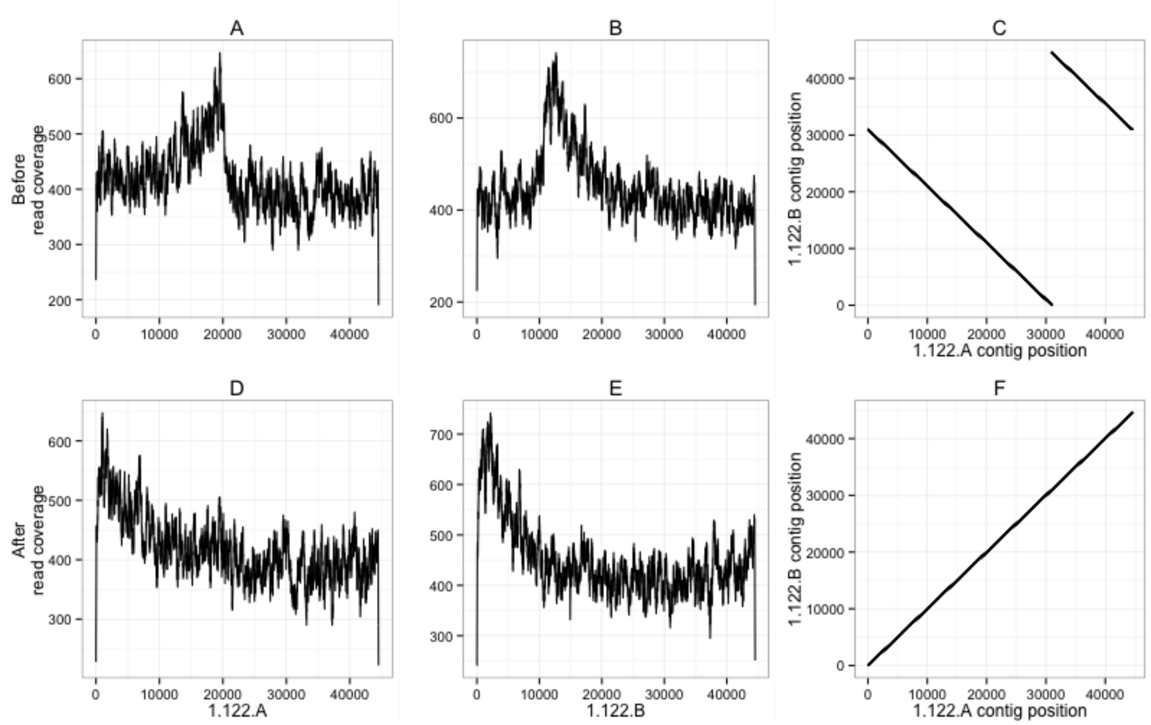

Supplementary Figure 1. Read coverage patterns for replicate viruses predicted to have headful-like packaging modes. Comparison of independent assemblies of two viruses representing sub-lineages of a single plaque isolate, before (**a** and **b**) and after (**d** and **e**) contig rearrangement; and dot plot comparison of assemblies for the same two viruses before (**c**) and after (**f**) contig rearrangement (see Supplementary Table 2 for rearrangement strategy).

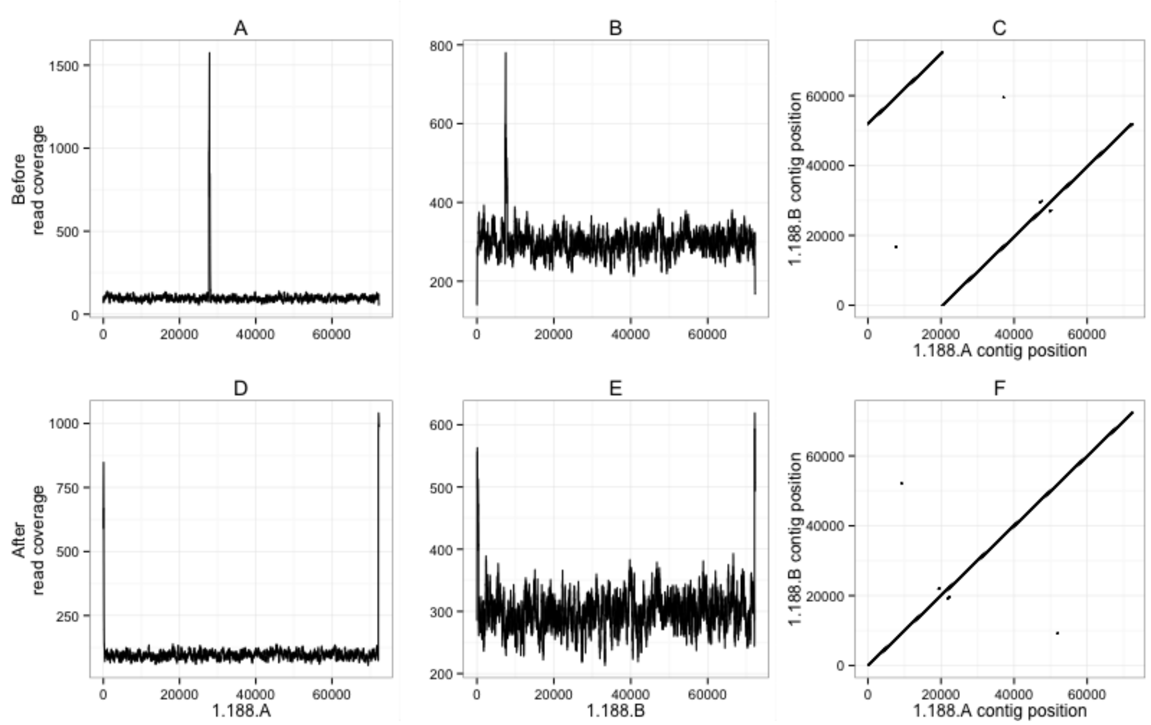

Supplementary Figure 2. Read coverage patterns for replicate viruses predicted to have genomes with terminal repeat sequences. Comparison of independent assemblies of two viruses representing sub-lineages of a single plaque isolate before (**a** and **b**) and after (**d** and **e**) contig rearrangement. Dot plot comparison of assemblies before (**c**) and after (**f**) rearrangement (see Supplementary Table 2 for rearrangement strategy).

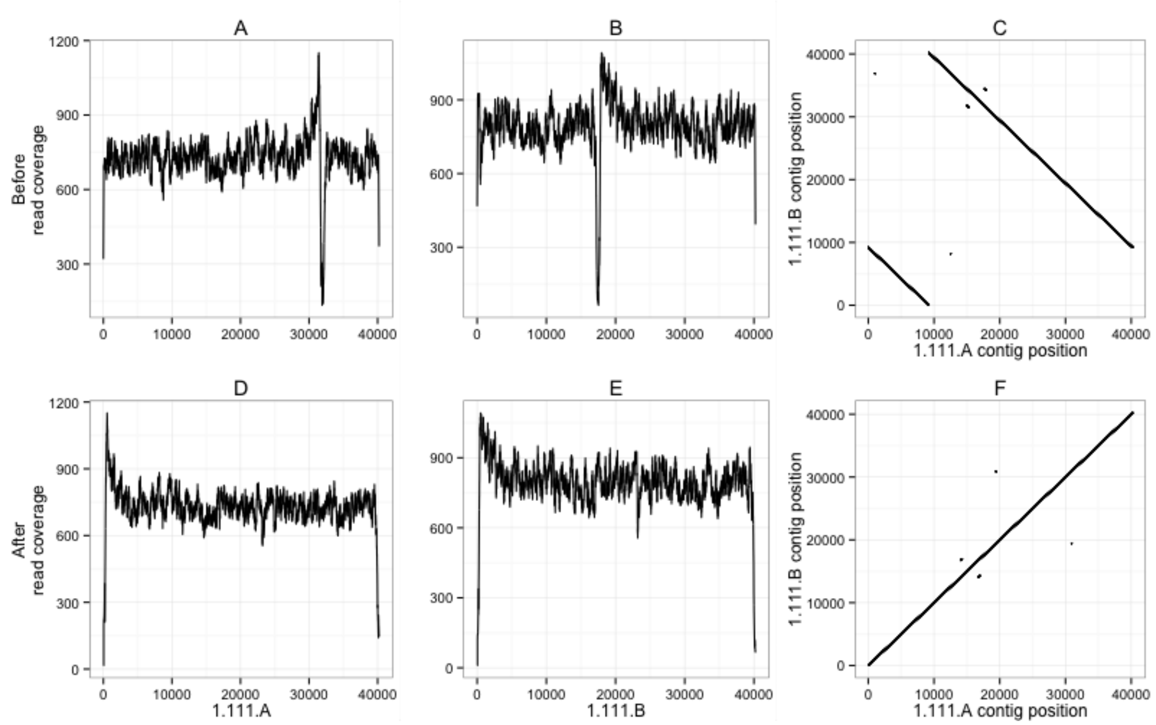

Supplementary Figure 3. Read coverage patterns for replicate viruses predicted to have genomes with terminal single-stranded cos-overhangs. Comparison of independent assemblies of two viruses representing sub-lineages of a single plaque isolate, before (**a** and **b**) and after (**d** and **e**) contig rearrangement; and dot plot comparison of assemblies before (**c**) and after (**f**) rearrangement (see Supplementary Table 2 for rearrangement strategy).

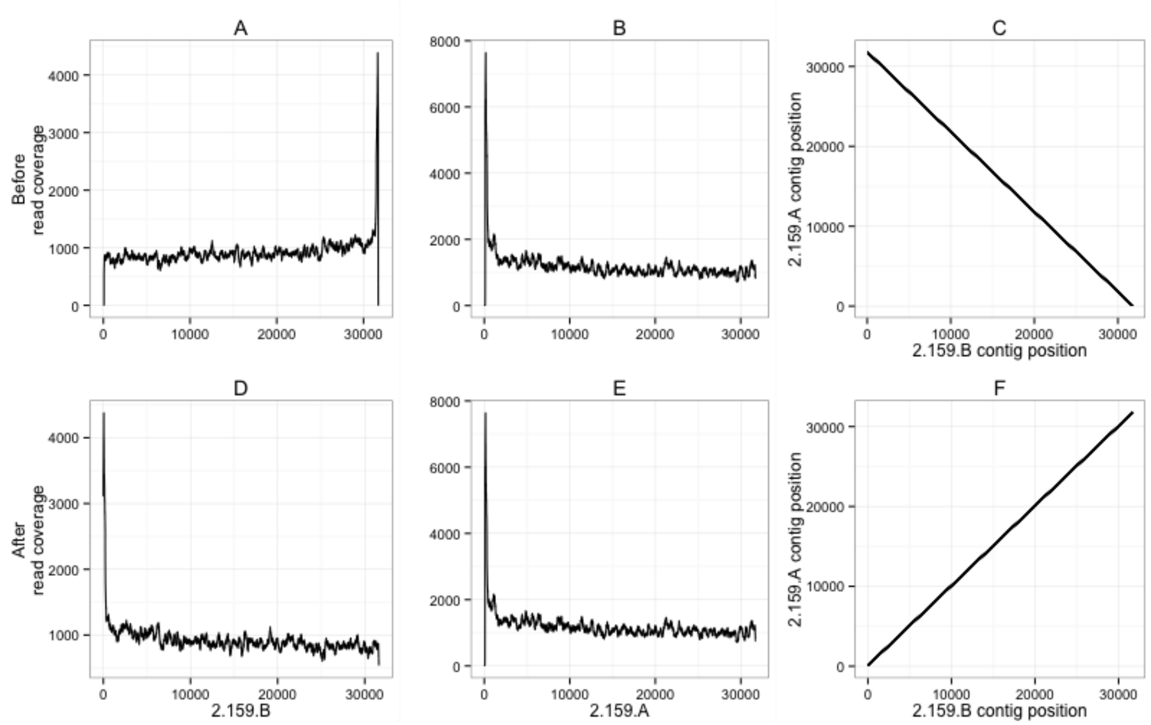

Supplementary Figure 4. Read coverage patterns for replicate viruses predicted to have Mu-like replication modes. Comparison of independent assemblies of two viruses representing sub-lineages of a single plaque isolate, before (**a** and **b**) and after (**d** and **e**) genome adjustment; and dot plot comparison of assemblies before (**c**) and after (**f**) rearrangement (see Methods for final assembly strategy).
